# Efficacy of pirfenidone for the treatment of pulmonary fibrosis: An updated systematic review protocol of randomized controlled trial

**DOI:** 10.1097/MD.0000000000015407

**Published:** 2019-04-26

**Authors:** Shu-Min Li, Yang Lin, Shan-Shan Liang

**Affiliations:** Department of Respiratory Medicine, First Affiliated Hospital of Jiamusi University, Jiamusi, China.

**Keywords:** efficacy, pirfenidone, pulmonary fibrosis, safety

## Abstract

**Background:**

This study will systematically assess the efficacy and safety of pirfenidone for the treatment of patients with pulmonary fibrosis (PF).

**Methods:**

We will search potential records from following literature sources from their inceptions to the present without language, and publication status limitations: Cochrane Library, EMBASE, PUBMED, the Cumulative Index to Nursing and Allied Health Literature, the Allied and Complementary Medicine Database, Chinese Biomedical Literature Database, and China National Knowledge Infrastructure. In addition, we also search grey literature sources, such as dissertations, conference proceedings, as well as the reference lists of included studies. All randomized controlled trials related to the pirfenidone for treating PF will be included. All the process of study selection, data extraction, and methodological evaluation will be carried out by 2 authors independently. The primary outcome comprises of all-cause-mortality, and lung function status, as measured by forced vital capacity. The secondary outcomes consist of 6-minute walk distance, progression-free survival, dyspnea, acute exacerbation, quality of life, and adverse events. Whenever possible, all results data will be pooled and meta-analysis will be performed.

**Results:**

This study will systematically assess the efficacy and safety of pirfenidone for the treatment of patients with PF.

**Conclusion:**

The findings of the present study will summarize most recent evidence of pirfenidone for PF.

**Ethics and dissemination:**

No individual data will be analyzed in this study, thus, no research ethics approval is required in this study. The findings of this study are expected to be disseminated in a peer-reviewed journal or conference presentations.

**PROSPERO registration number:**

PROSPERO CRD42019126958.

## 1 Introduction

Pulmonary fibrosis (PF) is a very tricky lung disorder,^[1,2]^ characterized by severe and progressive fibrosis of the interstitium of lung, which mainly manifests as exertional dyspnoea and cough.^[3–6]^ This condition is very fatal, causing the death of patients within 2 to 5 years from diagnosis.^[7–10]^ It has been reported that the 5-year survival rate of PF varies from 20% to 40% associated with PF,^[11]^ which is similar to non-small cell lung cancer and even worse than other cancers.^[12]^

Pirfenidone is an antifibrotic drug with anti-inflammatory properties, which is widely used for the treatment of PF.^[13–17]^ Although a most recent systematic review has been published in October 2016,^[18,19]^ it searched the database only up to the February 2016, and most importantly, several high-quality randomized controlled trails (RCTs) have been published after that.^[19,20–24]^ Thus, it is still very necessary to conduct an updated study to systematically summarize the latest evidence for further assessing the efficacy and safety of pirfenidone for the treatment of PF.

## 2 Methods

### 2.1 Objectives

This study systematically assesses the efficacy and safety of pirfenidone for patients with PF.

### 2.2 Study registration

This protocol has registered on PROSPERO (CRD42019126958). It has reported based on the guidelines of Preferred Reporting Items for Systematic Reviews and Meta-Analysis (PRISMA) Protocol statement.^[25]^

### 2.3 Eligibility criteria for study selection

#### 2.3.1 Types of studies

This study will include RCTs of pirfenidone for PF without language and publication status restrictions. Non-RCTs and quasi-RCTs will be excluded in this study.

#### 2.3.2 Types of patients

Patients with PF regardless race, gender, age or educational background will be included in this study.

#### 2.3.3 Types of interventions

In the experimental group, patients can receive any forms of pirfenidone monotherapy. In the control group, patients can undergo any treatments, except the pirfenidone therapy.

#### 2.3.4 Types of Outcomes

##### 2.3.4.1 Primary outcomes

All-cause-mortality;

Lung function status (as measured by forced vital capacity).

##### 2.3.4.2 Secondary outcomes

6-minute walk distance;

Progression-free survival;

Dyspnea (as assessed by University of California San Diego Shortness of Breath Questionnaire, or other instruments);

Acute exacerbation;

Quality of life (as evaluated 36-Item Short Form Health Survey, or other scales);

Adverse events (any expected or unexpected adverse events or reactions).

### 2.4 Search strategy for identification of studies

The following literature sources will be searched from their inceptions to the present without language, and publication status limitations: Cochrane Library, EMBASE, PUBMED, the Cumulative Index to Nursing and Allied Health Literature, the Allied and Complementary Medicine Database, Chinese Biomedical Literature Database, and China National Knowledge Infrastructure. Additionally, grey literature sources, including dissertations, conference proceedings, and reference lists of included studies will also be considered for inclusion. We have presented the detailed search strategy for PUBMED in Table 1. We have also used similar search strategy to other electronic databases.

**Table 1:**
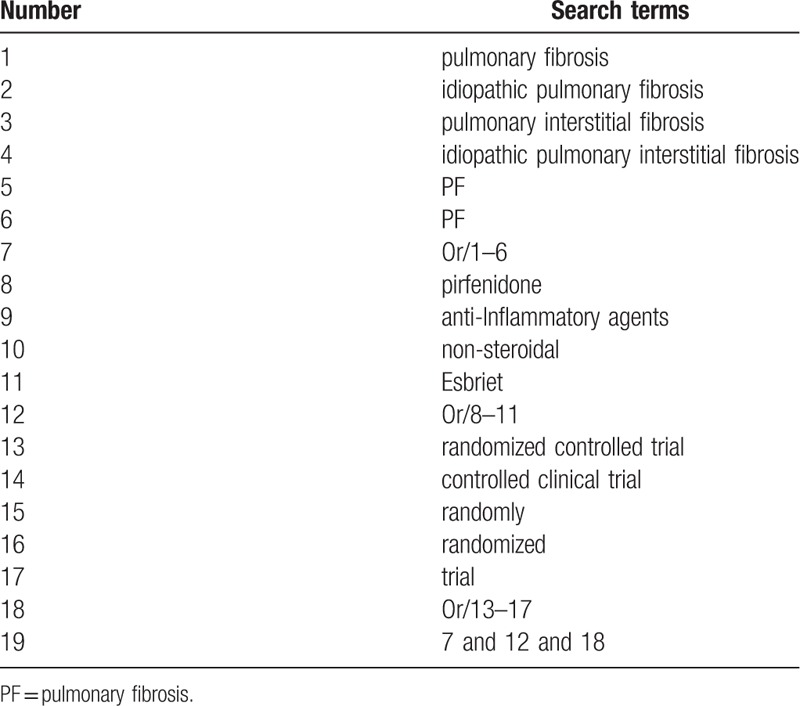
Search strategy used in PUBMED database.

### 2.5 Study selection

EndNote 7.0 software will be used to manage the searched records, and remove the duplicated literature. Two authors will independently review the title and abstract for each searched record to confirm eligible studies. Full texts will also be read if they can not be identified from titles and abstracts. All disagreements will be arbitrated by a third author through discussion. We will document each excluded study with clearly reason. The results of study selection will be presented in PRISMA flowchart.

### 2.6 Data extraction

Two authors will perform data extraction using a pre-designed data extraction sheet respectively. Any disagreements will be discussed with a third author. All essential information and data will be collected from each eligible trial for following information: basic information, patients, study methods, details of treatments, outcome measurements, adverse events, and any other relevant information.

### 2.7 Risk of bias assessment for eligible trials

Risk of bias for each eligible trail will be judged according to the standard criteria of Cochrane Risk of Bias Tool through 7 aspects. Each aspect will be further judged as 3 levels: high risk of bias, unclear risk of bias, and low risk of bias. Two authors will assess the risk of bias for each study respectively. All divisions regarding the risk of bias assessment will be solved by a third author through discussion

### 2.8 Measurement of treatment effect

Continuous data will be measured by using mean difference or standardized mean difference and 95% confidence intervals (CIs). Dichotomous data will be measured by using risk ratio and 95% CIs.

### 2.9 Missing data dealing with

We will try our best to contact primary authors of eligible studies to obtain missing or insufficient data. If we can not require those additional data, only available data will be analyzed. However, we will discuss its potential impacts.

### 2.10 Heterogeneity identification

In this study, *I*^*2*^ test will be used to identify heterogeneity among eligible studies. When *I*^*2*^ ≤50%, low heterogeneity is considered. When *I*^*2*^ >50%, significant heterogeneity is regarded, and subgroup analysis will be conducted.

### 2.11 Data synthesis

If low heterogeneity is identified, a fixed-effect model will be applied to pool the data and we will also carry out meta-analysis. If significant heterogeneity is detected, a random-effect model will be used to pool the data. Additionally, we will also carry out subgroup analysis to explore any possible causes that may account for significant heterogeneity. We will not pool the data, and will not perform meta-analysis if there is still significant heterogeneity after subgroup analysis. However, we will carry out a systematic narrative synthesis for the findings.

### 2.12 Subgroup analysis

We will operate subgroup analysis in accordance with different characteristics, interventions, and outcomes.

### 2.13 Sensitivity analysis

Sensitivity analysis will be performed to test the robustness and stability of the outcome results by removing low quality of eligible studies, and also the possible effects of missing data.

### 2.14 Reporting bias

We will carry out funnel plot^[26]^ and Egger regression test^[27]^ to identify any possible reporting bias if more than 10 eligible RCTs will be included in this study.

## 3 Discussion

The protocol of this study will apply rigorous methodology to identify and examine studies reporting the outcomes of pirfenidone for PF. Although most recent similar study has been reported in 2016,^[18]^ there were more than 6 high-quality RCTs after that.^[19,20–24]^ Thus, it is still very necessary to update and summarize latest evidence for assessing the efficacy and safety of pirfenidone for PF.

This study will summarize rigorous evidence of pirfenidone for PF across all published RCTs. The findings of this study will inform our understanding of the value of pirfenidone in treating PF outcomes. This evidence may also provide helpful evidence for clinical practice and health policy-makers for the treatment of PF.

## Author contributions

**Conceptualization:** Shu-Min Li, Yang Lin, Shan-Shan Liang.

**Data curation:** Shu-Min Li, Yang Lin, Shan-Shan Liang.

**Formal analysis:** Shu-Min Li, Shan-Shan Liang.

**Investigation:** Shu-Min Li.

**Methodology:** Yang Lin, Shan-Shan Liang.

**Project administration:** Shu-Min Li.

**Resources:** Shu-Min Li, Yang Lin, Shan-Shan Liang.

**Software:** Yang Lin, Shan-Shan Liang.

**Supervision:** Shu-Min Li.

**Validation:** Shu-Min Li, Shan-Shan Liang.

**Visualization:** Shu-Min Li.

**Writing – original draft:** Shu-Min Li, Yang Lin, Shan-Shan Liang.

**Writing – review & editing:** Shu-Min Li, Yang Lin, Shan-Shan Liang.
